# Demonstration of Lignin-to-Peroxidase Direct Electron Transfer: A TRANSIENT-STATE KINETICS, DIRECTED MUTAGENESIS, EPR, AND NMR STUDY*

**DOI:** 10.1074/jbc.M115.665919

**Published:** 2015-08-03

**Authors:** Verónica Sáez-Jiménez, Maria Camilla Baratto, Rebecca Pogni, Jorge Rencoret, Ana Gutiérrez, José Ignacio Santos, Angel T. Martínez, Francisco Javier Ruiz-Dueñas

**Affiliations:** From the ‡ Centro de Investigaciones Biológicas, CSIC, Ramiro de Maeztu 9, E-28040 Madrid, Spain,; the § Department of Biotechnology, Chemistry and Pharmacy, University of Siena, Via Aldo Moro, I-53100 Siena, Italy,; the ¶ Instituto de Recursos Naturales y Agrobiología de Sevilla, CSIC, P. O. Box 1052, E-41080 Seville, Spain, and; the ‖ NMR Facility, SGIKER, Universidad del País Vasco, UPV/EHU Donostia, 48940 Leioa, Bizkaia Spain

**Keywords:** electron paramagnetic resonance (EPR), enzyme kinetics, lignin degradation, nuclear magnetic resonance (NMR), peroxidase, HSQC NMR, lignosulfonate, transient-state kinetics, tryptophanyl radical, versatile peroxidase

## Abstract

Versatile peroxidase (VP) is a high redox-potential peroxidase of biotechnological interest that is able to oxidize phenolic and non-phenolic aromatics, Mn^2+^, and different dyes. The ability of VP from *Pleurotus eryngii* to oxidize water-soluble lignins (softwood and hardwood lignosulfonates) is demonstrated here by a combination of directed mutagenesis and spectroscopic techniques, among others. In addition, direct electron transfer between the peroxidase and the lignin macromolecule was kinetically characterized using stopped-flow spectrophotometry. VP variants were used to show that this reaction strongly depends on the presence of a solvent-exposed tryptophan residue (Trp-164). Moreover, the tryptophanyl radical detected by EPR spectroscopy of H_2_O_2_-activated VP (being absent from the W164S variant) was identified as catalytically active because it was reduced during lignosulfonate oxidation, resulting in the appearance of a lignin radical. The decrease of lignin fluorescence (excitation at 355 nm/emission at 400 nm) during VP treatment under steady-state conditions was accompanied by a decrease of the lignin (aromatic nuclei and side chains) signals in one-dimensional and two-dimensional NMR spectra, confirming the ligninolytic capabilities of the enzyme. Simultaneously, size-exclusion chromatography showed an increase of the molecular mass of the modified residual lignin, especially for the (low molecular mass) hardwood lignosulfonate, revealing that the oxidation products tend to recondense during the VP treatment. Finally, mutagenesis of selected residues neighboring Trp-164 resulted in improved apparent second-order rate constants for lignosulfonate reactions, revealing that changes in its protein environment (modifying the net negative charge and/or substrate accessibility/binding) can modulate the reactivity of the catalytic tryptophan.

## Teaser

**Background:** Ligninolytic peroxidases are often studied using simple model substrates.

**Results:** Reaction of versatile peroxidase with the lignin polymer is shown by a combination of directed mutagenesis, chromatographic, and spectroscopic techniques.

**Conclusion:** The main reaction implies direct electron transfer to a surface tryptophanyl radical responsible for lignin oxidation.

**Significance:** This is the first time that this central mechanistic aspect is shown for a ligninolytic peroxidase.

## Introduction

About 20% of the total carbon fixed in nature is incorporated into lignin. It is the only main component of biomass with an aromatic origin, and a valuable raw material as a potential source of aromatic simple chemicals and polymers (1). Moreover, due to the recalcitrant nature of the lignin polymer, its removal is a limiting step to access the plant polysaccharides (cellulose and hemicelluloses) as a sustainable industrial feedstock (2, 3). Recent studies suggest that some bacterial enzymes are involved in degradation of lignin or lignin decay products (4, 5). However, white-rot fungi are the main decayers of wood lignin in nature (6), acting through a battery of secreted oxidoreductases that contribute to a degradation process defined as an “enzymatic combustion” (7). Among these enzymes, high redox-potential peroxidases (of the lignin peroxidase (LiP),^5^ manganese peroxidase, and versatile peroxidase (VP) families) play a central role in lignin attack (8). The above is in agreement with the presence of ligninolytic peroxidase genes (of the above families) in the genomes of all typical white-rot (lignin-degrading) fungi sequenced to date (poor wood rotters excluded) and their absence from the genomes of all sequenced brown-rot (cellulose-degrading) fungi (9–11).

VP was first described in *Pleurotus eryngii* (12, 13), and it combines catalytic and structural properties of: (i) LiP, characterized by the presence of a surface tryptophan residue involved in oxidation of high redox-potential substrates (such as veratryl alcohol mediating lignin degradation by this enzyme); (ii) manganese peroxidase, characterized by the presence of a Mn-binding site at one of the heme propionates, where Mn^2+^ is oxidized to Mn^3+^ (a diffusible oxidizer acting on lignin phenolic units); and (iii) generic peroxidases, which (as described for plant peroxidases) oxidize low redox-potential compounds at the heme access channel (14). These hybrid properties allow VP to oxidize a variety of substrates (15, 16) that, as recently reported, also include synthetic lignin and model dimers (17).

**FIGURE 1.**
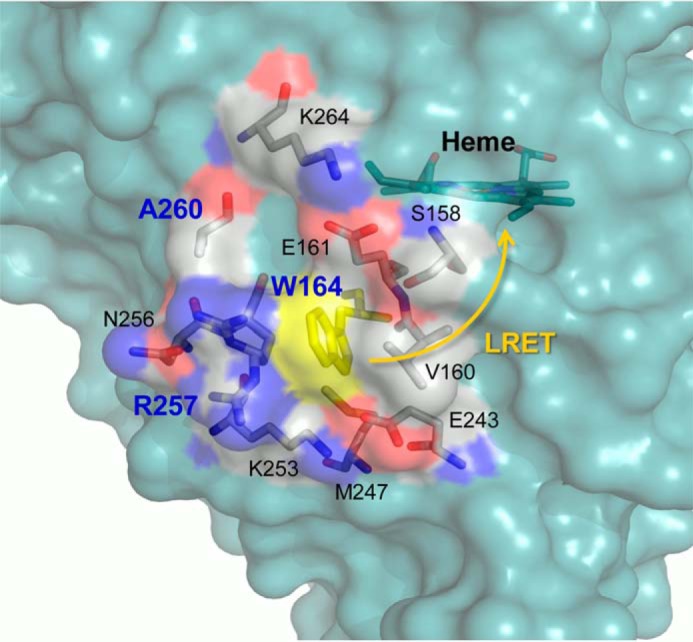
**Environment of the exposed catalytic tryptophan acting as starting point for LRET to heme in VP.** Trp-164, 10 neighbor residues (those changed by directed mutagenesis in *bold*), heme (all as Corey-Pauling-Koltun-colored sticks), and semitransparent protein surface (*blue*, except for the above tryptophan, in *yellow*, and the neighbor residues, in Corey-Pauling-Koltun colors) are shown

Ligninolytic peroxidases are of interest for enzymatic delignification processes, enabling the industrial use of plant polysaccharides, as well as for the transformation of lignin into added-value products (3). Due to the potential application of these peroxidases as industrial biocatalysts, an in-depth knowledge of the mechanisms underlying and modulating their catalytic properties is of the highest biotechnological importance. During a basic catalytic cycle, similar to that of other peroxidases, the VP resting state (containing a Fe^3+^ heme) is activated by H_2_O_2_ yielding Compound I (CI, an Fe^4+^=O·porphyrinyl radical complex). CI catalyzes a 1-electron substrate oxidation and is converted into Compound II (CII, which contains Fe^4+^=O). Through another 1-electron oxidation of a second substrate molecule, the enzyme resting state is recovered. In an extension of the above cycle, VP oxidizes high redox-potential compounds through an exposed catalytic tryptophan, which forms a radical (in both the CI and the CII transient states) on the surface of the protein through a long-range electron transfer (LRET) to the heme (18) (Fig. 1).

In this work, the ability of VP to act on the lignin polymer was investigated using water-soluble sulfonated lignins. Lignosulfonates are obtained through the sulfite pulping of wood (19), and have been commercialized for a range of applications (for example, agriculture; animal feed additives; dispersants; construction; and other important areas through companies such as Borregaard LignoTech). Representative softwood lignin, which contains monomethoxylated (guaiacyl) units, and hardwood lignin, which has both monomethoxylated and dimethoxylated (syringyl) units, were used in this study. The main aim of the work was to study the transient-state kinetics of VP electron abstraction from the above lignins, the role played by a putative catalytic tryptophan in this redox reaction (through the formation of a tryptophanyl free radical), and the modification of lignin during steady-state treatment of lignosulfonates with VP. With this purpose, a combination of site-directed mutagenesis, stopped-flow rapid spectrophotometry, fluorescence spectroscopy, size-exclusion chromatography (SEC), EPR, and NMR techniques was used.

## Experimental Procedures

### 

#### 

##### Enzyme Production

VP from *P. eryngii* and its W164S and R257A/A260F mutated variants (16, 20) were produced in *Escherichia coli* as reported elsewhere (21). In short, a pFLAG1 expression plasmid (International Biotechnologies Inc., Cambridge, UK) containing the mature protein-coding sequence of isoenzyme VPL2 (GenBank^TM^ AF007222) was transformed into *E. coli* W3110. The cells were grown in Terrific Broth until optical density ∼1. Then, protein expression was induced by 1 mm isopropyl-β-d-thiogalactopyranoside, and cells were further grown for 4 h. The apoprotein accumulated as inclusion bodies and was recovered in a solution of 8 m urea, 1 mm DTT, and 1 mm EDTA in 50 mm Tris-HCl (pH 8).

Subsequent *in vitro* reactivation was carried out overnight in a solution of 0.16 m urea,15 μm hemin, 5 mm CaCl_2_, 0.1 mm DTT, 0.5 mm oxidized glutathione, and 0.1 mg ml^−1^ VP protein in 20 mm Tris-HCl (pH 9.5). Finally, native VP and its variants were purified by anion-exchange chromatography (Resource Q column, GE Healthcare, Uppsala, Sweden) using a 0–0.3 m NaCl gradient (2 ml min^−1^, 20 min) in 10 mm sodium tartrate (pH 5.5) containing 1 mm CaCl_2_. The *R_z_* (*A*_410_/*A*_280_ ∼ 4) of the variants was indicative of the purity of the proteins. The UV-visible spectra were checked to confirm the correct folding and cofactor incorporation in the activated enzymes. Isopropyl-β-d-thiogalactopyranoside, DTT, hemin, and oxidized glutathione were from Sigma-Aldrich, and urea and EDTA were from Merck (Darmstadt, Germany).

##### Softwood and Hardwood Lignins

**FIGURE 2.**
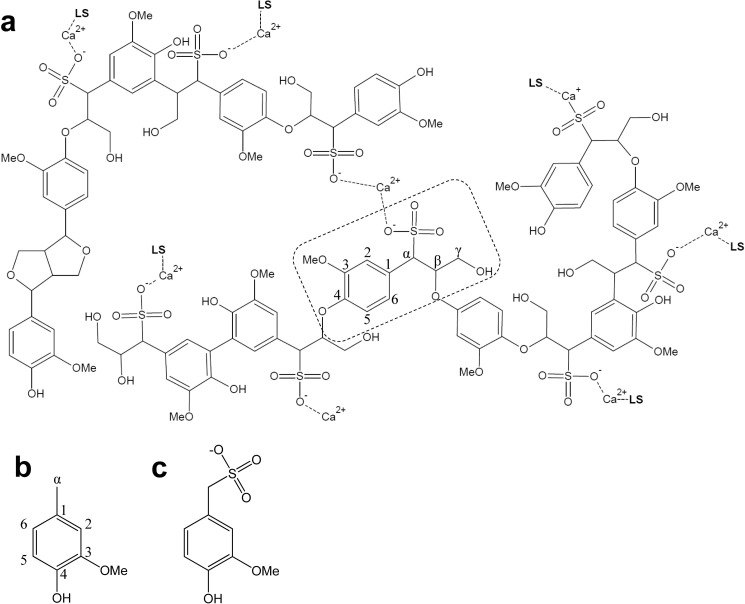
**Tentative structure for sulfonated softwood lignin (22) and simple model compounds.** *a*, structure of softwood lignosulfonate as Ca^2+^ salt (*LS*, lignosulfonate chains; *MeO*, methoxyl group). *b* and *c*, formulae of creosol (*b*), and α-sulfonated creosol (*c*). The basic phenylpropanoid unit is shown (*box*), and carbon labeling is indicated. See Fig. 8*e* for the main structures identified in softwood (*P. abies*) and hardwood (*E. grandis*) lignosulfonates

Two water-soluble sulfonated lignins were used in this study: softwood (*Picea abies*) lignosulfonate and hardwood (*Eucalyptus grandis*) lignosulfonate, both kindly provided by G. E. Fredheim (Borregaard AS, Sarpsborg, Norway). The lignosulfonate samples were first dialyzed in 10 mm EDTA, 50 mm Tris (pH 8) with the aim of removing Mn^2+^ traces (which reduce H_2_O_2_-activated VP), and then in Milli-Q water to remove buffer and EDTA. A model for softwood lignosulfonate chemical structure (22) is included in Fig. 2*a*, showing guaiacyl units linked by β-O-4′ alkyl-aryl ether and other minor bonds. Creosol (Sigma-Aldrich) (Fig. 2*b*) and α-sulfonated creosol, kindly provided by R. A. Lauten (Borregaard AS) (Fig. 2*c*), were used as simple models for normal and sulfonated lignin units in stopped-flow experiments.

##### Enzyme (Transient-state) Kinetics

Reduction reactions of CI and CII of wild-type recombinant (hereinafter native) VP and its W164S and R257A/A260F variants in 0.1 m tartrate (pH 3) by softwood and hardwood lignosulfonates, creosol, and α-sulfonated creosol were followed using a stopped-flow rapid spectrophotometry equipment (Bio-Logic, Claix, France) with a three-syringe module (SFM300) synchronized to a diode array detector (J&M, Essingen, Germany) and Bio-Kine software.

CI reduction was studied by mixing the enzyme (1 μm final concentration) with H_2_O_2_ (1 μm final concentration) for 0.6 s, resulting in CI formation. Next, substrates were added at different concentrations, and the reactions were followed at 416 nm (isosbestic point of VP CII and resting state). CII reduction was studied by mixing a solution of enzyme and ferrocyanide (both at 1 μm final concentration) with H_2_O_2_ at equimolar ratio. The solution was aged for 6 s, and CII formation was achieved. Then, different excess concentrations of substrate were added, and the reaction was followed at 406 nm (Soret maximum of resting VP). For comparison, the lignosulfonate concentrations in these and other experiments were referenced to the basic phenylpropanoid unit, shown in Fig. 2 (corresponding to 260 and 290 Da in the sulfonated softwood and hardwood lignins, respectively).

All kinetic traces exhibited single-exponential character from which pseudo-first-order rate constants (*k*_2obs_ and *k*_3obs_ for CI and CII reduction, respectively) were calculated. Plots of *k*_2obs_ *versus* substrate concentration were fitted to a linear or hyperbolic model from which apparent second-order rate constants (*k*_2app_) were obtained. Plots of native VP *k*_3obs_ *versus* substrate concentration were fitted to a Michaelis-Menten model from which *K_D_*_3_ (dissociation constant of the enzyme-substrate, VP-lignosulfonate, complex) and *k*_3_ (first-order rate constant) were obtained. The corresponding *k*_3app_(*k*_3_/*K_D_*_3_) rate constants were calculated with the equation: *k*_3obs_ = (*k*_3_/*K_D_*_3_)[S]/(1+[S]/*K_D_*_3_), where [S] indicates substrate concentration. R257A/A260F CII reduction plots were fitted to a sigmoid model from which *k*_3_ and *K*_0.5_ (substrate concentration resulting in 50% *k*_3_ that, in the present work, was considered equivalent to *K_D_*_3_) constants were obtained. To calculate the *k*_3app_ values, the following equation was used: *k*_3obs_ = *K_D_*_3_ (*k*_3_/*K_D_*_3_)[S]*^b^*/([S]*^b^*+*K_D_*_3_*^b^*), where *b* is the Hill coefficient. Finally, W164S CII reduction plots were fitted to a linear model from which *k*_3app_ values were determined.

##### EPR of VP Reactions with Lignin

EPR measurements were performed in solutions containing 0.16 mm VP, 1.3 mm H_2_O_2_, and 0 (1:8:0 ratio), 0.64 (1:8:4 ratio), or 1.92 mm (1:8:12 ratio) softwood lignosulfonate in 50 mm tartrate (pH 3). The reactions were initiated by the addition of H_2_O_2_ and stopped by immersion in liquid nitrogen after a few seconds. CW-X-band (9-GHz) EPR measurements were carried out with a E500 Elexsys series instrument (Bruker, Karlsruhe, Germany) using the Bruker ER 4122 SHQE cavity and an ESR900 helium continuous flow cryostat (Oxford Instruments, Abingdon, UK) at 40 K. Measurements with the W164S variant were performed as described above, at 9, 20, and 40 K. Spin quantification was performed by double-integration of the experimental EPR tryptophanyl radical signal as compared with the iron signal of the enzyme in the resting state. To calculate the percentage of lignin radical, the pure tryptophanyl radical signal was considered as our reference. The area (double integration) of the tryptophanyl radical signal, with the proper intensity, was subtracted from the area of the mixed signal including the tryptophanyl and lignin radicals (see “Results”).

##### Lignosulfonate Treatment under Steady-state Conditions (Fluorescence Monitoring)

Softwood and hardwood lignosulfonates (12 g/liter^−1^) were treated with VP (1.3 μm) and H_2_O_2_ (12.5 mm, final concentration, added continuously over 24 h with a syringe pump) in 50 mm phosphate (pH 5), at 25 °C, and samples were taken after different times (3, 12, and 24 h). Control treatments were performed under the same conditions but in the absence of enzyme. Although VP shows the highest activity at pH 3 (as used in stopped-flow experiments), the above long-term lignosulfonate treatments were performed at pH 5 to maintain the enzyme active during the whole incubation period. Changes in lignin fluorescence were monitored (excitation at 355 nm and emission at 400 nm) using a Fluorolog-3-221 instrument (Horiba Jobin Yvon, Longjumeau, France) at 25 °C. The lignin samples were diluted to 10 μg ml^−1^ in 2-methoxyethanol/water (2:1 v/v) before the fluorescence measurement.

##### SEC and GC-MS Analyses

Changes in the molecular mass distribution of the VP-treated softwood and hardwood lignosulfonates and controls (described above) were analyzed by SEC using a Superdex-75 column (HR-10/30, 3,000–70,000/100,000-Da range; GE Healthcare) with 0.1 m NaOH as the mobile phase, at a flow rate of 0.5 ml min^−1^. UV (280 nm), refraction index, and multi-angle laser light scattering detections were compared. Blue dextran (Serva, Heidelberg, Germany) was used to determine the exclusion volume of the column, and a kit of sulfonated polystyrene sodium salt standards with main peaks (Mp) in the 891–976,000-Da range (PSS, Mainz, Germany) was used for calibration and mass determination (*V_e_*/*V*_0_ *versus* Log(Mp), where *V_e_* and *V*_0_ are the elution and void volumes).

Low molecular mass compounds in the reaction mixtures and controls were investigated by GC-MS after liquid-liquid extraction with methyl *tert*-butyl ether (Merck, Darmstadt, Germany) at pH 2. The extracts, with and without *N*,*O*-bis(trimethylsilyl)-trifluoroacetamide (Sigma-Aldrich) derivatization, were analyzed using a GCMS-QP2010 Ultra instrument (Shimadzu Co., Kyoto, Japan) and a J&W capillary column (DB-5HT 30 m × 0.25-mm inner diameter, 0.10-μm film thickness). The oven was heated from 60 °C (1 min) to 350 °C (2 min) at 10 °C/min. The injector was set at 350 °C and the transfer line was kept at 300 °C. Helium was used as the carrier gas (1 ml min^−1^). Compounds were identified and quantified using authentic standards.

##### Lignin NMR

Samples after different times (3, 12, and 24 h) of softwood and hardwood lignosulfonate treatment and the corresponding controls (described above) were freeze-dried for NMR analyses. Solution NMR spectra, including ^13^C NMR and heteronuclear single quantum correlation (HSQC) two-dimensional NMR spectra, were recorded at 25 °C on an AVANCE III 500 MHz instrument (Bruker) equipped with a cryogenically cooled 5-mm TCI gradient probe with inverse geometry. The lignosulfonate samples (40 mg of initial weight, before treatments) were dissolved in 0.75 ml of deuterated DMSO-*d*_6_. The central solvent peak was used as the internal reference (at δ_C_/δ_H_ 39.5/2.49 ppm), and the other signals were normalized to the same intensity of the DMSO signals (because the same DMSO volume and the same initial amount of sample were used in all the cases).

The HSQC experiment used Bruker's “hsqcetgpsisp2.2” adiabatic pulse program with spectral widths from 0 to 10 ppm (5,000 Hz) and from 0 to 165 ppm (20,625 Hz) for the ^1^H and ^13^C dimensions. The number of transients was 64, and 256 time increments were always recorded in the ^13^C dimension. The ^1^*J*_CH_ used was 145 Hz. Processing used typical matched Gaussian apodization in the ^1^H dimension and squared cosine-bell apodization in the ^13^C dimension. Prior to Fourier transformation, the data matrices were zero-filled to 1024 points in the ^13^C dimension. Signals were assigned by literature comparison (23–28). In the aromatic region of the spectrum, the lignin C_2_-H_2_, C_5_-H_5_, and C_6_-H_6_ correlation signals were integrated to estimate the amount of lignins and the syringyl-to-guaiacyl ratio. In the aliphatic oxygenated region, the signals of methoxyls, as well as C_β_-H_β_ (or C_α_-H_α_) correlations in the side chains of sulfonated and non-sulfonated β-O-4′, phenylcoumaran, and resinol substructures, were also integrated. The intensity corrections introduced by the adiabatic pulse program permit us to refer the latter integrals to the previously obtained number of lignin units. Cross-polarization magic-angle spinning ^13^C NMR spectra of solid lignosulfonate samples were recorded for 9–72 h on a Bruker AVANCE III 400 using the standard pulse sequence, a time domain of 2 K, a spectral width of 41 Hz, a contact time of 1.5 ms, and an interpulse delay of 5 s.

## Results

### 

#### 

##### Transient-state Kinetics of Electron Abstraction from Lignin

**FIGURE 3.**
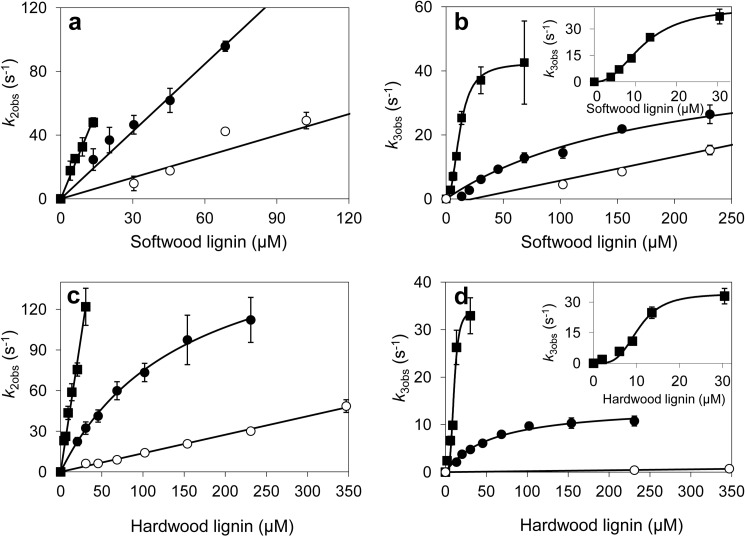
**Kinetics of reduction of CI (*a* and *c*) and CII (*b* and *d*) of native VP (●) and its W164S (○) and R257A/A260F (■) variants by softwood (*a* and *b*) and hardwood (*c* and *d*) lignosulfonates.** Stopped-flow reactions were carried out at 25 °C in 0.1 m tartrate (pH 3). The lignosulfonate concentrations in Figs. 3–5 refer to the basic phenylpropanoid unit, as explained under “Experimental Procedures.” Means and 95% confidence limits are shown. *Insets* show the R257A/A260F kinetic curves for a smaller concentration range. *Error bars* indicate means ± S.E.

**FIGURE 4.**
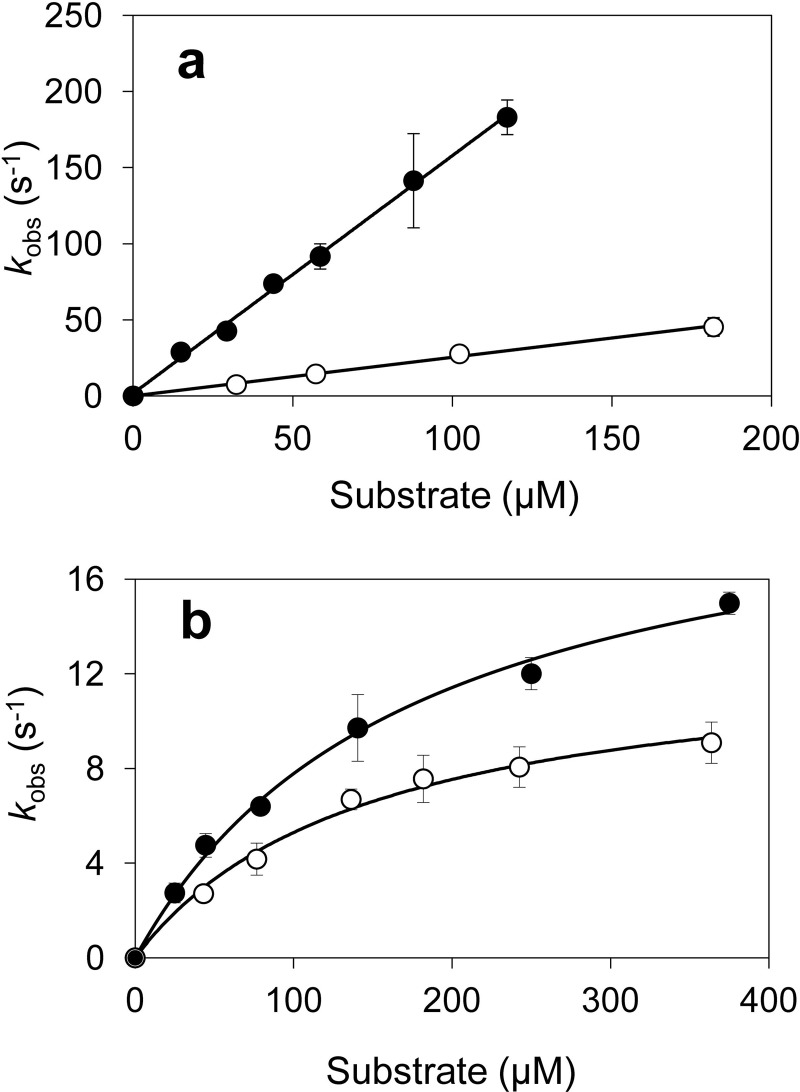
**Kinetics of VP CI (*a*) and CII (*b*) reduction by creosol (*black circles*) and the corresponding sulfonate derivative (*white circles*).** Stopped-flow reactions were carried out at 25 °C in 0.1 m tartrate (pH 3). Means and 95% confidence limits are shown. *Error bars* indicate means ± S.E.

The transient-state kinetic constants for lignosulfonate reaction with VP were obtained from stopped-flow measurements. Native VP CI/CII were able to react with lignin from both softwood and hardwood (Fig. 3, *top* and *bottom*, respectively), exhibiting similar apparent second-order rate constants (*k*_app_) (Table 1), although some differences were observed in the CII reduction *K_D_*_3_ and *k*_3_ values. The dissociation constant *K_D_*_3_ was 3.4-fold lower for hardwood lignosulfonate than for softwood lignosulfonate, indicating a higher affinity of the enzyme for the former lignin. A similar decrease was observed in the *k*_3_ constant for the hardwood lignosulfonate, and this was the reason for the similar efficiency in the oxidation of both lignins. The kinetics for reduction of VP CI/CII by creosol and α-sulfonated creosol were also examined (Fig. 4). Although CI seems to be more efficient at oxidizing creosol (1560 ± 35 s^−1^ mm^−1^) than its sulfonated counterpart (253 ± 8 s^−1^ mm^−1^), CII reduction (the limiting step in catalysis) showed similar efficiencies (122 ± 8 and 89 ± 8 s^−1^ mm^−1^, respectively) and *k*_3_ (21 ± 1 and 13 ± 1 s^−1^, respectively) and *K_D_*_3_ (175 ± 21 and 147 ± 22 μm, respectively) values for the two model substrates.

**TABLE 1:** **Transient-state kinetic constants for the reduction of CI and CII of the native VP and its W164S and R257A/A260F variants by softwood and hardwood lignosulfonates** Shown are first-order rate constants, *k*_3_ (s^−1^); equilibrium dissociation constants, *K_D_*_3_ (μm); and apparent second-order rate constants, *k*_2app_ and *k*_3app_ (s^−1^mm^−1^). Means and 95% confidence limits. The lignosulfonate concentrations refer to the basic sulfonated phenylpropanoid unit, as explained under “Experimental Procedures.”

	CI reduction		CII reduction					
	Softwood	Hardwood	Softwood			Hardwood		
	*k*_2app_	*k*_2app_	*k*_3_	*K_D_*_3_	*k*_3app_	*k*_3_	*K_D_*_3_	*k*_3app_
VP	1410 ± 30	1240 ± 50	48 ± 2	194 ± 21	250 ± 20	14 ± 1	57 ± 7	250 ± 20
W164S	660 ± 90	140 ± 3	—*^a^*	—*^a^*	70 ± 10	—*^a^*	—*^a^*	2 ± 0.2
R257A/A260F	3680 ± 150	4020 ± 130	40 ± 1	11 ± 1*^b^*	3540 ± 70	34 ± 3	10 ± 1*^b^*	3260 ± 240

*^a^* —, not determined because saturation was not reached.

*^b^ K*_0.5_ values (substrate concentration at which the velocity is half-maximal) obtained using the Hill equation and, in the present work, *K*_0.5_ was considered equivalent to *K_D_*_3_.

Besides native VP, two variants were tested for the oxidation of lignosulfonates (Fig. 3 and Table 1). In the W164S variant, the substitution of the putative catalytic tryptophan resulted in impaired oxidation of both lignosulfonates. The strongest effect of the W164S mutation was observed in the case of the hardwood lignosulfonate, where the *k*_2app_ and (rate-limiting) *k*_3app_ values experienced 9- and 125-fold decreases, respectively, with respect to the native enzyme. The remaining reduction of W164S CI and CII is attributed to the minor phenolic units present in the softwood (2.1%) and hardwood (1.4%) lignosulfonates.^6^

The second VP variant (R257A/A260F) harbors two surface mutations near Trp-164 (Fig. 1). Interestingly, this variant showed enhanced transient-state kinetic constants (Fig. 3, and Table 1), as well as sigmoidal kinetics for CII reduction (Fig. 3. *b* and *d*, *insets*). A 13–14-fold improved (rate-limiting) *k*_3app_ was observed, revealing higher efficiency in the oxidation of both lignosulfonates. This was due to 17- and 6-fold decreases of *K_D_*_3_ for softwood and hardwood lignosulfonates, respectively, indicative of a higher affinity. CI reduction was also improved, although the increases in *k*_2app_ were not as high (Fig. 3, *a* and *c*, and Table 1).

##### EPR Detection of VP and Lignin Radicals

**FIGURE 5.**
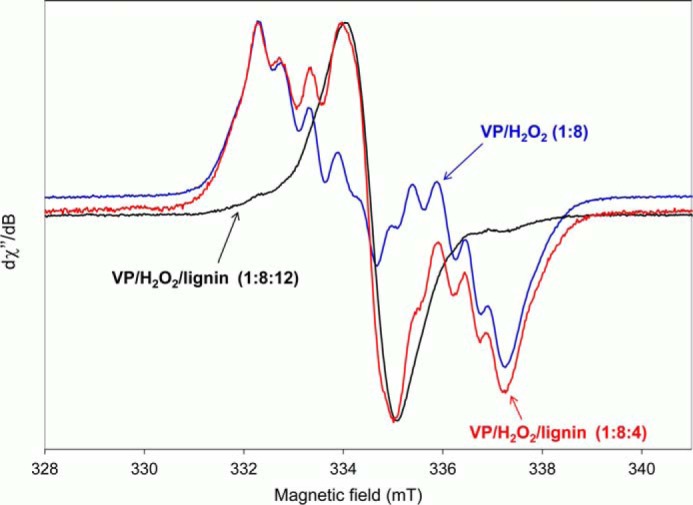
**EPR spectra of the reactions of VP with H_2_O_2_ (at molar ratio 1:8), and of VP with H_2_O_2_ and softwood lignosulfonate at two different molar ratios (1:8:4 or 1:8:12).** All spectra were recorded at 40 K, 9.394 GHz, 1-milliwatt microwave power, and 0.2 mT modulation amplitude, a few seconds after mixing. Intensity-normalized spectra are shown (integration values for the tryptophanyl and lignin radical signals in the original spectra are provided under “Results”).

EPR spectra of VP reactions with different concentrations of softwood lignosulfonate were obtained (after enzyme activation by H_2_O_2_). In the absence of reducing substrate, a protein radical was observed (Fig. 5, *blue line*), whose abundance from EPR signal integration (referenced to the iron signal in the resting state enzyme) was estimated to be ∼0.25 spin/heme. Under the above conditions (*T* = 40 K), no protein radicals were detected for the W164S variant, whereas a characteristic porphyrinyl radical (18), centered at g = 2.00 (±0.01) with a Δ*H*_pp_ ∼0.22 mT, was observed (at *T* < 30 K), whose intensity quickly declined when the acquisition temperature increased from 9 to 40 K.

When a small amount of lignin was added (VP/H_2_O_2_/lignin ratio of 1:8:4), a mixed radical signal centered on both the protein and the lignin was detected (Fig. 5, *red line*), whose lignin radical only represented ∼0.9% of the tryptophanyl radical signal intensity in the previous VP spectrum without lignin. When a higher amount of lignin was added (VP/H_2_O_2_/lignin ratio of 1:8:12), the protein radical signal was replaced by the intense lignin radical signal (Fig. 5, *black line*), which represented ∼35% of the intensity of the pure tryptophanyl signal in the VP spectrum. In the spectrum acquired with the highest lignin content, the residual protein radical contribution was visible on the wings of the lignin radical signal.

The protein radical signal (Fig. 5, *blue line*) is characterized by a large doublet splitting centered at g = 2.0027 (± 0.0001), with well resolved sub-splittings on each of the two components. This signal has been previously assigned to a tryptophanyl neutral radical (Trp-164) (16, 29) formed through LRET to the H_2_O_2_-activated heme. On the other hand, the lignin radical (Fig. 5, *black line*) is characterized by a single line at g = 2.0043 (± 0.0001) with a Δ*H*_pp_ ∼0.1 mT. This radical was stable for several hours, and could be detected even at 298 K.

##### Fluorescence Changes during the VP Treatments

**FIGURE 6.**
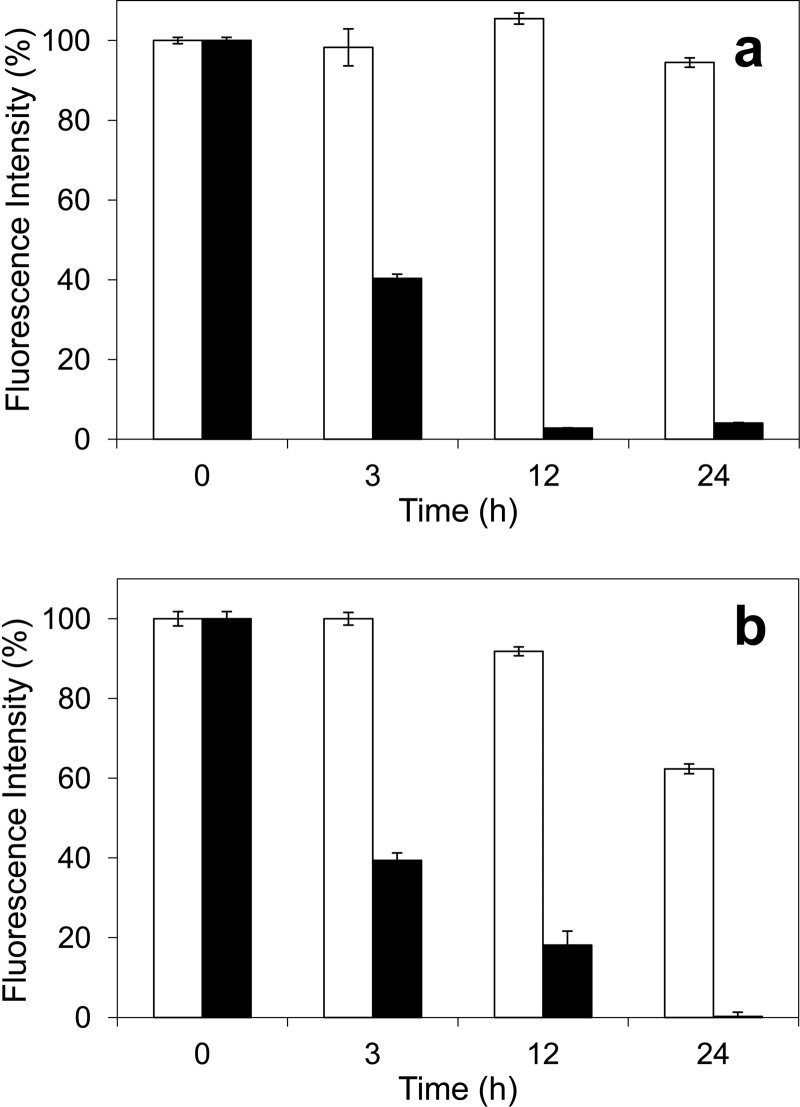
**Relative fluorescence of softwood (*a*) and hardwood (*b*) lignosulfonates during 24-h treatment with VP and H_2_O_2_ (*black bars*) and the corresponding controls without enzyme (*white bars*).** Changes of lignosulfonate (12 g/liter^−1^) fluorescence during treatment with VP (1.3 μm) and H_2_O_2_ (12.5 mm) in 50 mm phosphate (pH 5) were monitored (excitation at 355 nm/emission at 400 nm) after different time periods (3, 12, and 24 h). Means and 95% confidence limits are shown. *Error bars* indicate means ± S.E.

Changes in lignin fluorescence are easily detectable during steady-state treatments of lignosulfonates with VP. As shown in Fig. 6, a decrease of the fluorescence intensity to 40% (as compared with the initial intensity) was observed after 3 h of incubation of softwood and hardwood lignosulfonates with VP and H_2_O_2_. In addition, after 12 and 24 h of the enzymatic treatment of softwood lignin, the fluorescence was practically undetectable. In the case of the hardwood lignosulfonate, the fluorescence intensity decreased to a 20% after a 12-h treatment and was mostly inexistent after 24 h.

##### Changes of Lignin Molecular Mass

**FIGURE 7.**
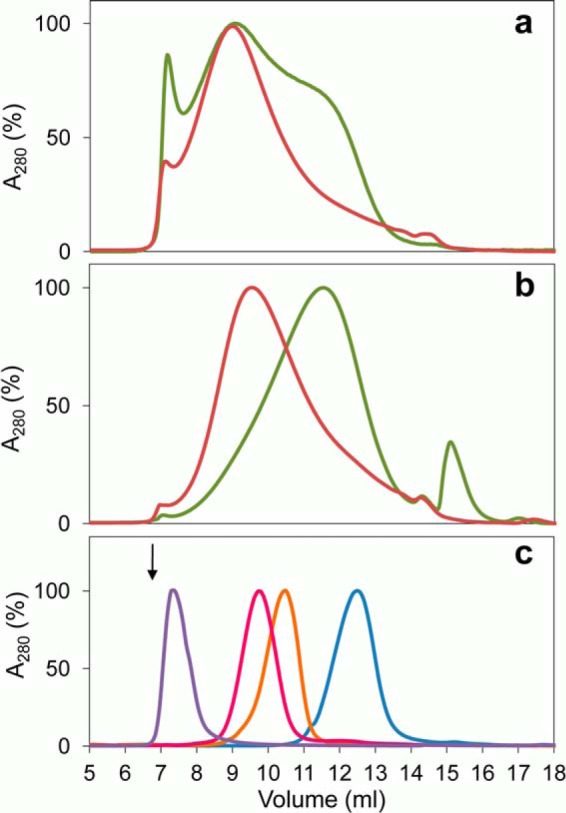
**Molecular mass distribution of VP-treated and control softwood (*a*) and hardwood (*b*) lignosulfonates, and sulfonated polystyrene standards (*c*).** Lignosulfonate samples (12 g/liter^−1^) after a 24-h treatment with VP (1.2 μm) and H_2_O_2_ (9.5 mm) (*red lines*), and the corresponding controls without enzyme (*green lines*), were analyzed in a Superdex-75 column using 0.15 m NaOH as eluent (0.5 ml min^−1^) and detection at 280 nm. Sulfonated polystyrenes (Mp 78,400, 29,500, 10,200, and 4,210 Da, from *left* to *right*) were used as molecular mass standards (*arrow* shows the blue dextran elution volume) (*c*).

Fig. 7 shows the molecular mass distribution profiles of softwood (Fig. 7*a*) and hardwood (Fig. 7*b*) lignosulfonates after 24-h treatment with VP (*red curve*) and control treatment without enzyme (*green curve*) in a Superdex-75 column (with 0.1 m NaOH as eluent, and 280-nm detection).

Using sulfonated polystyrene standards (Mp 78,400, 29,500, 10,200, and 4,210 Da) (Fig. 7*c*), we estimated Mp of ∼32,000 and ∼5,500 Da for the control softwood and hardwood lignosulfonates, respectively. The softwood lignosulfonate had a broader distribution with a large shoulder at ∼11.7-ml elution volume (∼6,800 Da) and a small excluded fraction. For hardwood lignosulfonate, a small peak was observed near the column total volume. Similar profiles were observed when the refraction index and multi-angle laser light scattering detectors were used, with the only exception of the low molecular mass peak that was detected only with the UV-visible detector (suggesting simple compounds absorbing at 280 nm).

The enzymatic treatment resulted in a net increase of the average molecular mass of the hardwood lignosulfonate (from Mp of ∼5,500 to ∼20,000 Da). For the softwood lignosulfonate, the Mp was not significantly modified, but the large shoulder disappeared (most probably being incorporated into the Mp of ∼33,000 Da). Simultaneously, the small excluded fraction in softwood lignosulfonate nearly disappeared, suggesting some depolymerization activity. Finally, the low molecular mass peak present in the control hardwood lignosulfonate was not observed after the enzymatic treatment.

The VP-treated lignosulfonates (and controls) were extracted with methyl *tert-*butyl ether and analyzed by GC-MS, but no relevant low molecular mass compounds derived from lignin could be identified. Only 2,6-dimethoxy-*p*-benzoquinone, a product from peroxidase oxidation of syringic acid (30) present in the control sample, was found after VP treatment of the hardwood lignosulfonate, albeit with very low abundance.

##### NMR Analyses of Lignosulfonates after VP Treatment

**FIGURE 8.**
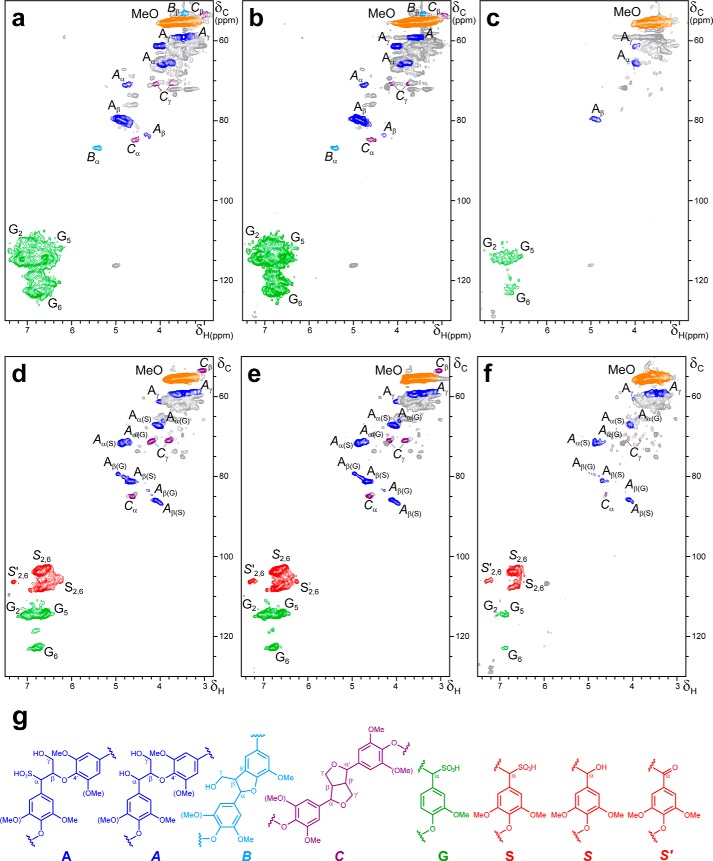
**HSQC spectra of softwood (*a–c*) and hardwood (*d–f*) lignosulfonates after 3-h (*b* and *e*) and 24-h (*c* and *f*) treatment with VP/H_2_O_2_, as compared with control without enzyme (*a* and *d*), and formulae of the main structures identified (*g*).** Signals correspond to ^13^C-^1^H correlations at the different positions of lignin normal/α-oxidized/α-sulfonated syringyl units (*red signals*), guaiacyl units (*green signals*), α-sulfonated/non-sulfonated side chains in β-O-4′ (*blue signals*), phenylcoumaran (*cyan signals*), and pinoresinol (*purple signals*) substructures, and methoxyls (*orange signals*) (*gray, unassigned signals*). Signals of β-O-4′ substructures with a second guaiacyl or syringyl unit could be identified. The same amount of sample (40 mg before treatment) and DMSO-*d*_6_ (0.75 ml) were used for all the spectra in Figs. 8 and 9, which were normalized to the same intensity of the DMSO signal (not shown) for comparison. List of signals (δ_C_/δ_H_ ppm): 53.2/3.46, C_β_/H_β_ in phenylcoumarans (***B*_β_**); 53.4/3.00, C_β_/H_β_ in resinols (***C*_β_**); 55.5/3.66, C/H in methoxyls (**MeO**); 59.4/3.4 and 3.72, C_γ_-H_γ_ in β-*O*-4′ (***A*_γ_**); 61.1/4.00, C_γ_-H_γ_ in sulfonated β-*O*-4′ (**A_γ_**); 65.6/3.93, C_α_/H_α_ in sulfonated β-*O*-4′ linked to a G unit (**A_α(G)_**); 67.2/4.02, C_α_/H_α_ in sulfonated β-*O*-4′ linked to an S unit (**A_α(S)_**); 70.8/4.16 and 3.77, C_γ_-H_γ_ in β-β′ resinols (***C*_γ_**); 71.1/4.72, C_α_/H_α_ in β-*O*-4′ linked to a G unit (***A*_α(G)_**); 71.5/4.85, C_α_/H_α_ in β-*O*-4′ linked to an S unit (***A*_α(S)_**); 79.3/4.91, C_β_/H_β_ in sulfonated β-*O*-4′ linked to a G unit (**A_β(G)_**); 80.9/4.67, C_β_/H_β_ in sulfonated β-*O*-4′ linked to an S unit (**A_β(S)_**); 83.3/4.24, C_β_/H_β_ in β-*O*-4′ linked to a G unit (***A*_β(G)_**); 84.9/4.59, C_α_/H_α_ in β-β′ resinols (***C*_α_**); 85.7/4.08, C_β_/H_β_ in β-*O*-4′ linked to an S unit (***A*_β(S)_**); 86.7/5.41, C_α_/H_α_ in phenylcoumarans (***B*_α_**); 103.8/6.68, C_2_/H_2_ and C_6_/H_6_ in syringyl units (***S*_2,6_**); 106.2/7.29, C_2_/H_2_ and C_6_/H_6_ in α-oxidized syringyl units (***S*′_2,6_**); 108.0/6.68, C_2_/H_2_ and C_6_/H_6_ in sulfonated syringyl units (**S_2,6_**); 114.0/6.60 and 114.3/6.87, C_2_/H_2_ and C_5_/H_5_ in guaiacyl units (**G_2_/G_5_**); and 122.8/6.75, C_6_/H_6_ in guaiacyl units (**G_6_**) (minor, and largely overlapping, signals of C_2_/H_2_, C_5_/H_5_, and C_6_/H_6_ correlations in non-sulfonated guaiacyl units would appear at 110.7/6.93, 114.2/6.65, and 118.6/6.79 ppm, respectively; not shown).

The chemical modification of lignin during the above VP treatments was analyzed by one-dimensional and two-dimensional NMR (the main structures identified are indicated in Fig. 8*g*). The HSQC spectrum of the control softwood lignosulfonate (Fig. 8*a*) shows the C_2_-H_2_, C_5_-H_5_, and C_6_-H_6_ aromatic correlations of the lignin guaiacyl units (**G**, *green signals*). In the aliphatic-oxygenated region, the C_α_-H_α_, C_β_-H_β_, and C_γ_-H_γ_ correlations of the lignin side chains forming inter-unit linkages in the main sulfonated (**A**) and minor non-sulfonated (***A***) β-O-4′ substructures (*blue signals*) were found. Small signals of phenylcoumarans (***B***, *cyan signals*) and pinoresinols (***C***, *purple signals*), whose side chains were not sulfonated due to C_α_ ether bond, and the prevalent methoxyl correlations (*orange signal*) were also observed. The spectrum of the control hardwood lignosulfonate (Fig. 8*d*) includes the above signals, with the only exception of phenylcoumaran signals, plus those of α-sulfonated (**S**), and non-sulfonated normal (***S***) and α-oxidized (***S*′**) syringyl units (*red signals*). Moreover, those β-O-4′ substructures including a second guaiacyl or syringyl unit could be discriminated. Another difference between the two lignins is the similar amount of sulfonated (**A**) and non-sulfonated (***A***) β-O-4′ substructures in the hardwood lignosulfonate, whereas only minor non-sulfonated β-O-4′ substructures (***A***) were found in the softwood lignosulfonate (and aromatic signals of α-sulfonated units were small and overlapped with those of sulfonated units, see the legend for Fig. 8).

During VP treatment of lignosulfonates, the signals of the different side-chain linkages (**A**, ***A***, ***B***, and ***C***) and aromatic (**G**, **S**, ***S***, and ***S*′**) lignin units (Fig. 8*g*) decreased, as shown in the 3-h (Fig. 8, *b* and *e*) and especially 24-h treatment spectra (Fig. 8, *c* and *f*), and some changes in their relative abundances were also observed. The similar decrease of both signal types resulted in only slightly modified numbers of inter-unit linkages per aromatic unit. However, the methoxyl numbers increased (up to 2.5- and 3.5-fold for hardwood and softwood lignosulfonates, respectively), suggesting the formation of non-aromatic methoxyl-containing (*e.g.* muconate type) structures. This was accompanied by the formation of α-oxidized syringyl units, whose relative abundance (with respect to total syringyl units) was 2- and 4-fold higher after 3- and 24-h treatments, respectively, in the hardwood lignosulfonate (where the S/G ratio also increased).

Concerning lignin side-chain signals, only those of the (main) sulfonated β-O-4′ substructures (**A_α_**, **A_β_**_,_ and **A_γ_**) remained after a 24-h treatment of the softwood lignosulfonate, and those of phenylcoumaran, resinol, and non-sulfonated β-O-4′ side chains were not detected. In contrast, those of sulfonated (**A**) and non-sulfonated (***A***) β-O-4′ and resinol (***C***) side chains were observed after a 24-h treatment of the hardwood lignosulfonate, albeit with low intensities. No changes in the aromatic/aliphatic HSQC signals were observed in the control treatment without enzyme.

**FIGURE 9.**
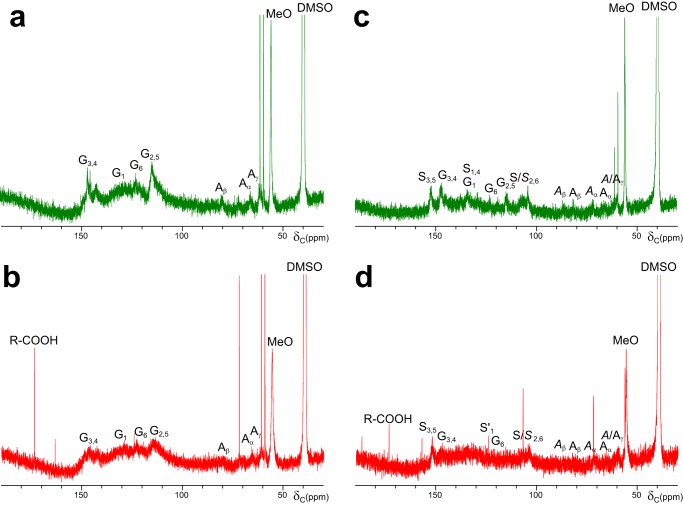
**^13^C NMR spectra of softwood (*a* and *b*) and hardwood (*c* and *d*) lignosulfonates after a 24-h treatment with VP/H_2_O_2_ (*b* and *d*), as compared with control without enzyme (*a* and *c*).** Signals of protonated (**G_2_**, **G_5_**, **G_6_**, and **S/*S*_2,6_**) and quaternary (**G_1_**, **G_3_**, and **G_4_**, and **S_1_**, **S_3_**, **S_4_**, and **S_5_**) carbons in guaiacyl and syringyl lignin units; α/β carbons in β-O-4′ linked sulfonated (**A_α_** and **A_β_**) and non-sulfonated (***A*_α_** and ***A*_β_**) lignin side chains; and methoxyls (**MeO**) are shown, together with a carboxyl (R-COOH) signal. Two sharp extra signals, at 59.2 and 61.0 ppm (one of them also observed in the HSQC spectra), most probably come from the buffer in lignosulfonate dialysis (before use), and the other in the treated samples remained to be assigned. List of quaternary carbon signals (δ_C_ ppm): 134, C_1_ in guaiacyl units (**G_1_**) and C_1,4_ in syringyl units (**S_1,4_**); 147, C_3,4_ in guaiacyl units (**G_3,4_**); and 152, C_3,5_ in syringyl units (**S_3,5_**) (see Fig. 8 legend for δ_C_ of protonated carbons).

Because only protonated carbons appear in the HSQC spectra, solution ^13^C NMR analyses were also performed. These spectra revealed that the VP treatment (Fig. 9, *b* and *d*) decreased not only the G_2_, G_5_, G_6_, and S/*S*_2,6_ signals, but also those of quaternary G_1_, G_3_, G_4_, S_1_, S_3,5_, and S_4_ carbons, as compared with the corresponding controls (Fig. 9, *a* and *c*). The minor C_α_, C_β_, and C_γ_ signals from the main β-O-4′ substructures (A/*A*_α/β/γ_) in the control spectra (Fig. 9, *a* and *c*) were barely detectable after the enzymatic treatment (Fig. 9, *b* and *d*). Moreover, a carboxyl signal (R-COOH) was observed in the spectra of the treated lignosulfonates, with the highest intensity in the softwood lignosulfonate spectrum. The above results were confirmed by solid-state cross-polarization magic-angle spinning ^13^C NMR spectra (not shown) that, despite their lower resolution, clearly showed a decrease of the different carbon types in lignin.

## Discussion

### 

#### 

##### Lignin oxidation by VP

*Phanerochaete chrysosporium* LiP was the first enzyme whose ability to depolymerize lignin and cleave non-phenolic lignin model dimers was reported (31, 32). More recently, a similar lignin-degrading ability was established for VP (17). In the present study, VP was demonstrated to directly oxidize lignin, and the electron transfer between the polymer and the enzyme was kinetically characterized for the first time in a peroxidase under transient-state conditions and assigned to the presence of a radical-forming tryptophan residue (VP Trp-164).

When comparing lignin oxidation with the oxidation of simple compounds, it is interesting to notice that the (rate-limiting) reduction of CII by lignosulfonates (250 s^−1^ mm^−1^) is (2-fold) better than found for creosol, and much better than the catalytic efficiencies for veratryl alcohol (∼60-fold higher) (16) and 3,4,5-trimethoxybenzyl alcohol (∼500-fold higher) (data not shown). The above suggests that lignins, at least water-soluble lignosulfonates, are more efficiently oxidized by VP than simple aromatics. It is interesting to know whether sulfonation, in addition to rendering lignin soluble, affects the oxidizability of its aromatic units by modifying the VP binding or turnover rate. With this purpose, creosol and its α-sulfonated form were compared as VP substrates. Although these models differ (in their phenolic nature and side-chain size) from the corresponding lignosulfonate units, the kinetic constants for CII reduction by sulfonated creosol were in the same range of those obtained for the softwood lignin. Moreover, α-sulfonation does not significantly modify oxidation of aromatic compounds (and most probably lignin) by VP because (rate-limiting) CII reduction by creosol was only slightly better than found for its sulfonated counterpart.

Two-dimensional NMR, in HSQC or similar experiments (in combination with ^13^C NMR), represents the state-of-the-art technique for the structural analysis of the lignin polymer (23, 33, 34). During the lignin treatment with VP, a progressive decrease in the intensity of the signals of the lignin aromatic units and side chains was observed, which correlated with the decrease of sample fluorescence. Lignin fluorescence is a known phenomenon (35, 36), and its decrease has been reported during lignosulfonate treatment with the laccase-mediator system (27). The progressive degradation of the different lignin structures was better shown in the HSQC spectra, but could also be observed in solution and solid-state ^13^C NMR for both protonated and quaternary carbons. Moreover, a carboxyl signal appeared in the ^13^C NMR spectra after the VP treatment.

Concomitantly with the lignin degradation shown by the fluorescence and NMR analyses, a net repolymerization of the oxidation products was shown by SEC, which was especially significant for the lower molecular mass hardwood lignosulfonate. This repolymerization would result from new diaryl C–C or ether substructures formed by aromatic radical condensation, as reported for lignosulfonate treatment with the lignin-degrading laccase-mediator system (27). It is interesting that in the VP reactions with lignosulfonates, no veratryl alcohol was added, as in related experiments (17), and despite this, significant lignin degradation was produced.

##### Role of VP Trp-164

It is assumed that ligninolytic peroxidases oxidize lignin (and other high redox-potential compounds) at a solvent-exposed residue, generally a tryptophan, susceptible to form a protein-based radical through LRET to heme (3, 14, 37). The involvement of VP Trp-164 in the oxidation of high redox compounds, such as veratryl alcohol and Reactive Black 5, has been demonstrated using the W164S variant that has no activity on these substrates (16, 18). However, the implication of Trp-164 in lignin oxidation and, more specifically, in the electron transfer from the polymer to the enzyme has not been demonstrated until now.

The kinetic results obtained for the lignosulfonate reaction with the W164S variant showed that *k*_2app_ and *k*_3app_ decreased to different extents. The residual activity is attributed to minor phenolic units in lignosulfonates (27) that would be oxidized at the heme access channel, as reported for oxidation of simple phenols by VP (38). Although electron transfer between synthetic lignin (dehydrogenation polymer) and *P. chrysosporium* LiP had been previously shown (39), here this transfer is directly related to the presence of the catalytic tryptophan (VP Trp-164, equivalent to LiP Trp-171).

The formation of a tryptophanyl neutral radical centered in Trp-164 has been reported after VP activation by H_2_O_2_ (16, 29) and supported by the absence of such a radical in the W164S spectrum, whereas a porphyrinyl radical signal was observed at 9 K, in agreement with previous studies (18). The EPR signal of this tryptophanyl neutral radical is mixed with a lignin radical signal when VP is incubated with a low concentration of lignosulfonate. Interestingly, when a higher concentration was used, the lignin radical signal predominated, and the tryptophanyl signal mostly disappeared. Therefore, we concluded that the Trp-164 radical is active on lignin because its reduction was observed concomitantly to the formation of a lignin radical, as found for veratryl alcohol oxidation by LiP (37) and VP (data not shown).

An in-depth analysis of the lignosulfonate radical detected by EPR could not be carried out because no hyperfine structure was observed. However, the g-value and the width of the signal are coherent with a radical formed by oxidation/hydrogen removal from a hydroxyl group of the substrate, in agreement with previously reported data (40–42). It has been shown that the treatment of kraft lignin with VP leads to the formation of phenoxy radicals (40), and the same has been reported for laccase and horseradish peroxidase (41). On the other hand, the long half-life of the lignin radical formed by VP can be due to the fact that the radicals are immobilized in the lignin matrix, rather than to a low reactivity (42).

##### Importance of the Trp-164 Environment

Although the solvent-exposed tryptophanyl radical is responsible for the oxidation of high redox-potential compounds, variations in the radical environment modulate its redox potential and stability (43), as well as the substrate binding. This is most probably responsible for differences between VP and LiP catalysis (20). In this way, LiP is able to oxidize veratryl alcohol more efficiently than VP does. This ability has been attributed to the acidic Trp-171 environment in *P. chrysosporium* LiP, which would facilitate the stabilization of the veratryl alcohol cation radical (44). On the other hand, whereas VP is able to oxidize Reactive Black 5 directly, LiP needs the presence of mediators to oxidize this anionic substrate, which would better bind to the more open and less acidic tryptophan environment of VP.

In this context, the R257A/A260F variant was designed to study in which way residues of the catalytic tryptophan environment could modulate the kinetic properties of VP (20). This variant showed sigmoidal plots for CII reduction, as well as improved transient-state kinetic constants for lignosulfonates. Sigmoidal plots in steady-state oxidation of some VP substrates have been related to the existence of two oxidation sites (corresponding to the catalytic Trp-164 and the heme access channel) (38). However, taking into account the position of the two mutations, as well as the molecular size of lignosulfonates, the sigmoidal plots obtained here suggest the existence of two different lignosulfonate binding poses in the environment of the catalytic tryptophan. Similar results were obtained for reduction of CII of the R257A/A260F variant by sulfonated Reactive Black 5 (20).

More interestingly, the R257A/A260F variant showed enhanced kinetic constants for both softwood and hardwood lignosulfonates, with 13–14-fold increased *k*_3app_. Similar improvements have been reported for veratryl alcohol oxidation (20) with final transient-state constants in the same order of those described for LiP (45). In this variant, Ala-260 was substituted by a phenylalanine, the residue at the homologous position in *P. chrysosporium* LiP that has been implicated in substrate binding by aromatic-aromatic interactions with veratryl alcohol oxidation (45). In the same way, VP interaction with lignin would be improved by this mutation, as suggested by the lower *K_D_*_3_ found for the R257A/A260F VP variant. On the other hand, the removal of basic Arg-257 would result in a more acidic catalytic tryptophan environment (as found in LiP) and would enhance VP reaction with lignin via aromatic cation radicals (37). In conclusion, the results from lignosulfonate oxidation by R257A/A260F VP show that the efficiency of the electron transfer from the polymer to the catalytic tryptophan depends on its surface environment and can be improved by modifying it.

##### Concluding Remarks

**FIGURE 10.**
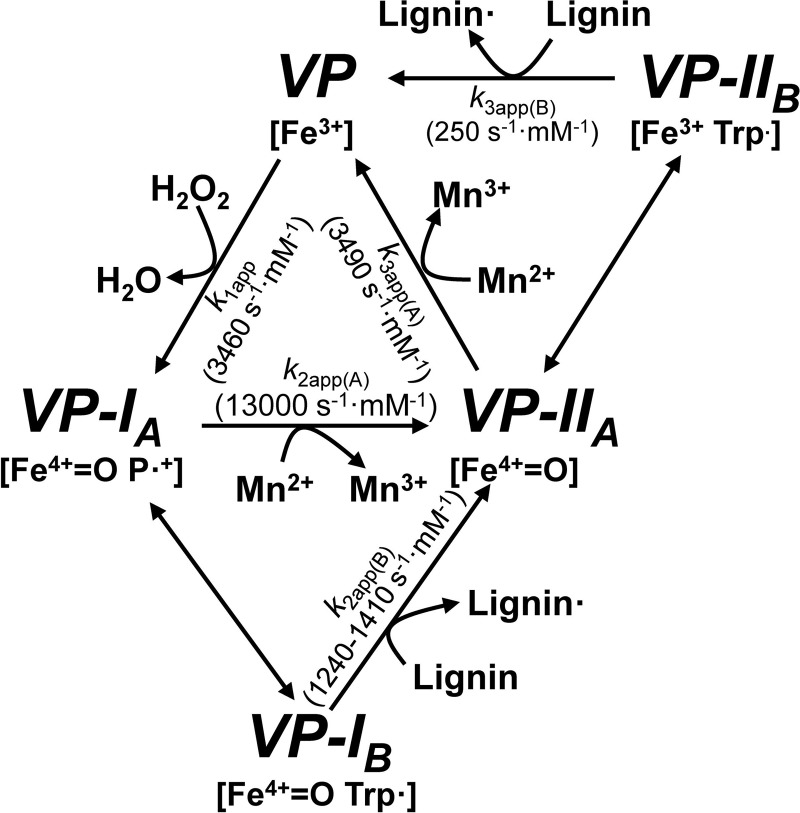
**VP catalytic cycle.** Shown is a scheme for VP catalytic cycle displaying resting state (Fe^3+^) activation by H_2_O_2_ and lignin oxidation by a tryptophanyl radical (VP-I_B_ and VP-II_B_) formed by one electron transfer from Trp-164 to VP-I_A_ (Fe^IV^=O·porphyrinyl radical, P^•+^, complex) and VP-II_A_ (Fe^IV^=O) heme. In contrast, Mn^2+^ is directly oxidized by VP-I_A_ and VP-II_A_. Other VP substrates, like phenols (including the lignin phenolic units) and dyes, can be oxidized both at the heme access channel (by VP-I_A_ and VP-II_A_) and at the catalytic tryptophan (by VP-I_B_ and VP-II_B_) (38) (not shown for simplicity). The above porphyrinyl radical was experimentally observed in the EPR spectrum of the peroxide-activated W164S variant (at 9 K), whereas the tryptophanyl radical was observed in VP spectra acquired at 40 K. The transient state (apparent second-order) rate constants for reactions with sulfonated lignins can be overestimated because some reaction is also produced at the heme channel (by VP-I_A_ and VP-II_A_), probably involving the minor phenolic units in lignin, as shown in Table 1 (W164S variant). The H_2_O_2_ and Mn^2+^ rate constants are taken from previous studies (16, 46). No constants are provided for the pass of VP-I_A_ and VP-II_A_ to VP-I_B_ and VP-II_B_, respectively, because electron deficiency is shared between the two redox centers (18).

The ability of VP to abstract electrons from softwood and hardwood lignin was demonstrated by stopped-flow spectrophotometry, using water-soluble lignosulfonates as substrate. This ability is related to the presence of Trp-164, as shown by site-directed mutagenesis. Moreover, EPR experiments detected the Trp-164 radical and demonstrated that it is catalytically active oxidizing lignin, as summarized in the enzyme cycle presented in Fig. 10. Improvements in the VP transient-state reactions with lignosulfonates, after the R257A/A260F double mutation, revealed the importance of the Trp-164 environment in the VP activity on lignin. Finally, NMR, fluorescence, and SEC analyses confirmed degradation and residual lignin repolymerization tendency during lignosulfonate treatment with VP under steady-state conditions.

## Author Contributions

V. S.-J. and F. J. R.-D performed most of the experimental biochemical work, and the biochemical data analysis. M. C. B. and R. P. contributed EPR experiments. J. R. and A. G. contributed solution-state NMR and GC-MS analyses. J. I. S. contributed solid-state NMR analysis. All authors participated in the interpretation and discussion of results. V. S.-J., F. J. R.-D, A. T. M., and R. P. contributed data integration and writing.

## Footnotes

6 G. E. Fredheim, personal communication.

## Footnotes

5 The abbreviations used are:

LiP: lignin peroxidase
CI: Compound I
CII: Compound II
HSQC: heteronuclear single-quantum correlation
LRET: long-range electron transfer
Mp: main peak
SEC: size-exclusion chromatography
VP: versatile peroxidase
DMSO: dimethyl sulfoxide
mT: milliteslas.
